# Thanatogenomic Investigation of the Hydroxymethylome and Mitochondrial Genome of Cadaveric Cardiomyocytes: Proposal for a Proof-of-Concept Study

**DOI:** 10.2196/17241

**Published:** 2020-03-05

**Authors:** Nerissa Naidoo, Gurjyot Bajwa, Ruthwik Duvuru, Yajnavalka Banerjee

**Affiliations:** 1 Department of Basic Medical Sciences Mohammed Bin Rashid University of Medicine and Health Sciences Dubai United Arab Emirates; 2 Heart and Vascular Institute Cleveland Clinic Abu Dhabi Al Maryah Island, Abu Dhabi United Arab Emirates; 3 Center for Medical Education University of Dundee Dundee United Kingdom

**Keywords:** epigenomics, mitochondrial genome, hypertrophic cardiomyopathy, undergraduate medical education

## Abstract

**Background:**

Cardiovascular disease (CVD) remains the leading cause of death in the United Arab Emirates (UAE). One of the common CVDs is hypertrophic cardiomyopathy (HCM). Recent studies conducted in heart cells of mice have shown that this condition involves a chemical modification called hydroxymethylation of the DNA of heart cells.

**Objective:**

Objectives of the proposed research are to profile the distribution of 5-hydroxymethylation in the cardiomyocyte (CMC) genome of cadaveric cardiac tissue and cardiac biopsy specimens; to compare the hydroxymethylome of cadaveric CMCs with that of cardiac biopsy specimens from HCM patients and/or cardiac transplant patients (control) undergoing cardiac catheterization; to histologically appraise sarcomere distribution and mitochondrial morphology of CMCs in the presence of HCM; to correlate the mitochondrial genome with the HCM phenotype; and to integrate anatomy with biochemistry and genetics into the instructional design of HCM in the core medical curriculum at Mohammed Bin Rashid University of Medicine and Health Sciences (MBRU).

**Methods:**

Normal and hypertrophic heart specimens will be obtained from 8 whole-body cadavers (2/8, 25% control and 6/8, 75% HCM). Myocardial biopsy specimens will be obtained from cardiothoracic and transplant units at the Cleveland Clinic in Abu Dhabi, UAE. As this is a proof-of-concept study, we plan to recruit 5 patients with HCM, where HCM has been diagnosed according to the guidelines of the 2014 European Society of Cardiology Guidelines. Patients with valvular heart disease, history of myocarditis, regular alcohol consumption, or cardiotoxic chemotherapy will be excluded. The control biopsy specimens will be obtained from patients who had received heart transplants. Three investigational approaches will then be employed: (1) gross anatomical evaluation, (2) histological analysis, and (3) profiling and analysis of the hydroxymethylome. These investigations will be pursued with minor modifications, if required, to the standard protocols and in accordance with institutional policy. The objective associated with the education of health professionals will be addressed through a strategy based on Graham’s knowledge translation model.

**Results:**

This study is at the protocol-development stage. The validated questionnaires have been identified in relation to the objectives. The MBRU and the Cleveland Clinic Abu Dhabi Institutional Review Board (IRB) are reviewing this study. Further clarification and information can be obtained from the MBRU IRB. There is funding in place for this study (MBRU-CM-RG2019-08). Currently, we are in the process of standardizing the protocols with respect to the various molecular techniques to be employed during the course of the study. The total duration of the proposed research is 24 months, with a provision for 6 months of a no-cost extension.

**Conclusions:**

The spectrum of CVDs has recently received significant focus from the public health sector in the UAE. HCM is a common familial heart disease, contributing to the sudden increase in the mortality rate of young Emiratis in the UAE. Incorporating artificial intelligence into the identification of epigenetic risk factors associated with HCM will promote accurate diagnosis and lead to the development of improved management plans, hence, positive patient outcomes. Furthermore, integration of these findings into the instructional design of undergraduate, postgraduate, and continuous professional development medical curricula will further contribute to the body of knowledge regarding HCM.

**International Registered Report Identifier (IRRID):**

PRR1-10.2196/17241

## Introduction

### Background

**Figure 1:**
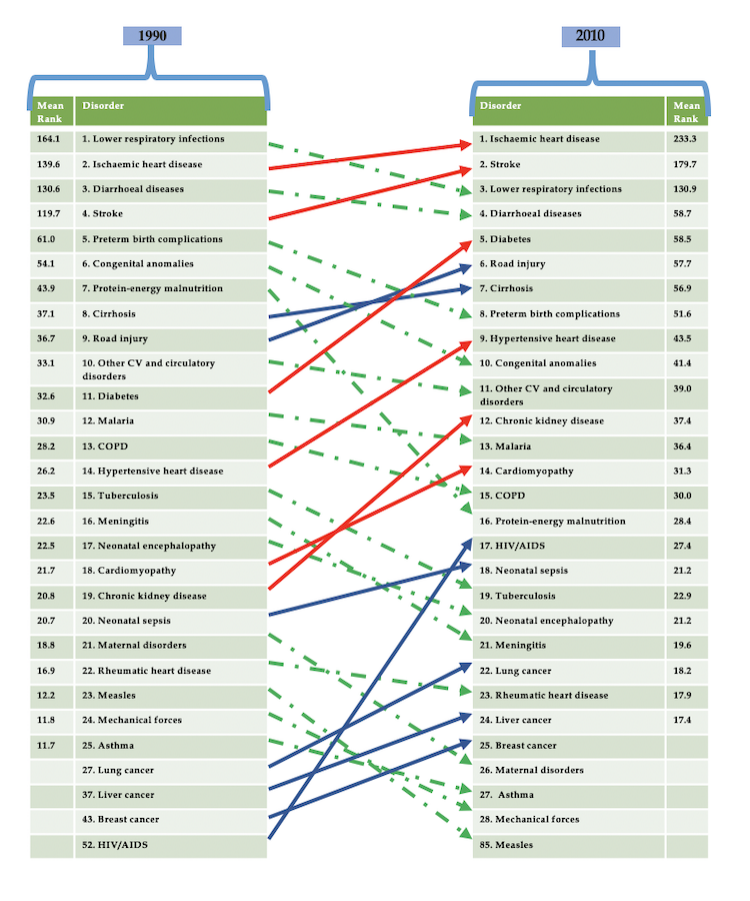
Top 25 causes of death in the Arab population in 1990 and 2010 (mean rank). A comparative analysis is provided. Solid blue lines indicate the elevation in rank for a condition or factor responsible for causing death in the Arab population from 1990 to 2010. Green dotted lines indicate a fall in rank of a condition or factor responsible causing death in the Arab population from 1990 to 2010. Red solid lines indicate the elevation or fall in rank of the factors contributing to cardiometabolic syndrome (CMS) in causing death from 1990 to 2010 (note: all the factors contributing to CMS are elevated in rank when compared between 1990 and 2010). Redrawn with modifications from Mokdad et al, 2014. COPD: chronic obstructive pulmonary disease; CV: cardiovascular.

**Figure 2:**
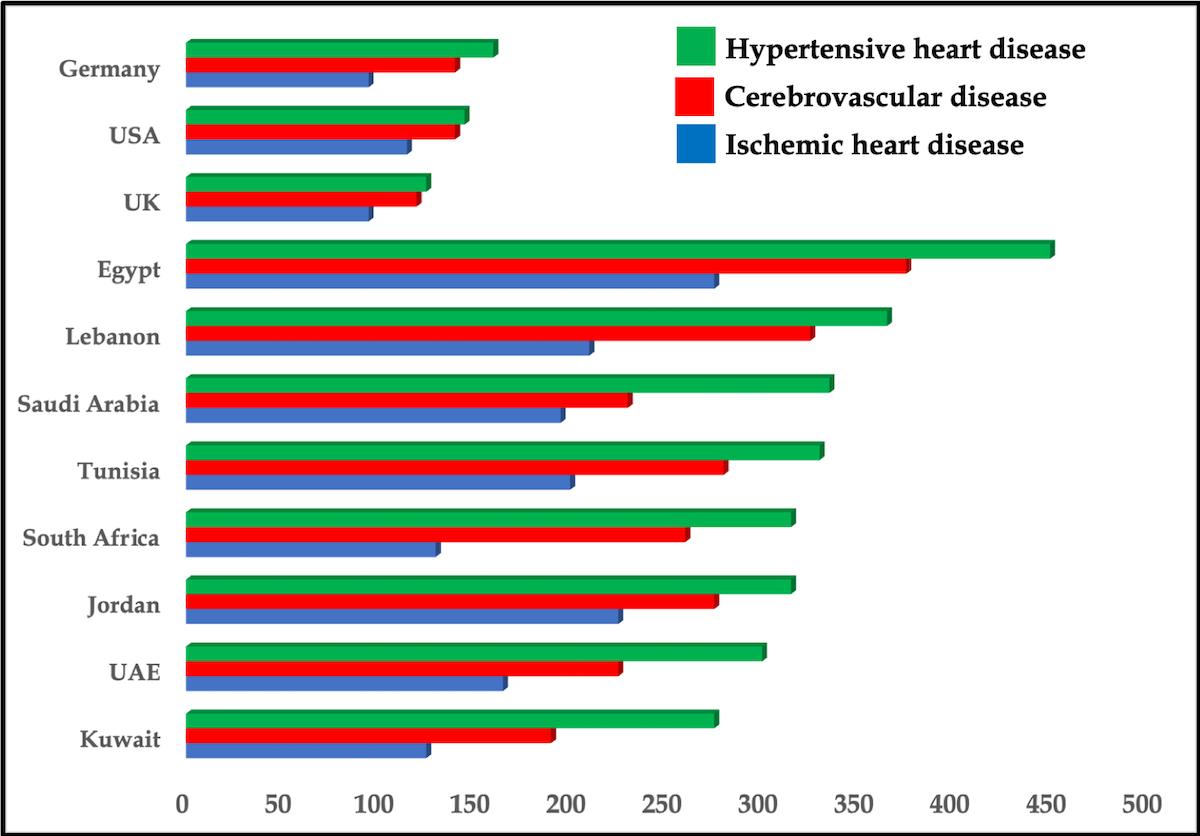
Death rates (ie, number of deaths per 100,000 people) from vascular diseases in selected countries in Africa and the Middle East compared to western countries. Drawn from data presented by the World Health Organization. UAE: United Arab Emirates.

Geographically, the Arab world is comprised of 22 countries from North Africa to Western Asia—Algeria, Egypt, Bahrain, Comoros, Djibouti, Iraq, Jordan, Saudi Arabia, Kuwait, Lebanon, Libya, Mauritania, Morocco, Oman, occupied Palestinian territory, Qatar, Yemen, Somalia, Sudan (including South Sudan), Syria, Tunisia, and the United Arab Emirates (UAE). These countries comprise the members of the League of Arab States. Each country has an inimitable set of historical, geopolitical, social, cultural, and economic characteristics, which define its public health systems and the burden of disease and injury. In 2014, Mokdad et al assessed the burden of disease and injuries in the 22 Arab countries in 1990, 2005, and 2010, employing data and methods from the Global Burden of Diseases, Injuries, and Risk Factors Study 2010 [1,2]. Interestingly, the study showed that since 1990, premature death and disability caused by communicable, newborn, nutritional, and maternal disorders, with the exception of HIV/AIDS, has decreased in the Arab world with a stark increase in ischemic heart disease, which contributed to 14.3% of deaths (see Figure 1) [1-3]. In addition, if one compares age-standardized mortality rates registered by the World Health Organization for specific countries in the region [4], a marked augmentation of cardiovascular deaths in most countries from the Middle East is observed, including in the UAE, when compared with data from comparator western countries (ie, the United Kingdom, Germany, and the United States); these deaths are predominantly from ischemic heart disease and hypertensive heart disease (see Figure 2).

Hence, with recent public health concerns focused on the high prevalence of cardiovascular diseases (CVDs) in the UAE, and with the sudden increase in deaths of relatively young Emiratis from CVD and associated disorders, the UAE National Service has implemented routine screening of all recruits for heart disease [5]. Interestingly, hypertrophic cardiomyopathy (HCM) has been identified as one of the four maladies contributing to the escalation in the mortality rate of young Emiratis, although the detailed statistics are still pending [5].

This necessitates that the molecular mechanism underlying HCM be elucidated, such that novel management and treatment strategies targeting the disorder can be identified and designed. Additionally, it is imperative that the concept of *genomics education of physicians* be integrated into the medical curriculum in the UAE, such that Emirati medical students can present findings based on correlation with known clinical information about the patients’ diseases and traits.

This is pivotal in light of the revelation that HCM is the most common familial heart disease with vast genetic heterogeneity. Two decades of rigorous investigation have described the vast and intimidating heterogeneity of the HCM substrate. Early reports of seven mutations in one gene—the myosin heavy chain beta isoform (MYH7) [6,7]—have now expanded to 11 or more causative genes with over 1400 mutations, expressed primarily or exclusively in the heart. These genes encode thick and thin myofilament proteins of the sarcomere or contiguous Z-disc. Mutations in several additional sarcomere, or calcium-handling, genes have been proposed, but with less evidence supporting pathogenicity [8]. Also, HCMs show remarkable variability in their age of onset, phenotypic presentation, and clinical course [9], alluding to the fact that disease mechanisms must exist that modify the occurrence and progression of HCM, either by genetic or epigenetic factors that may interact with environmental stimuli and external influences. According to Frey et al [10], HCM develops in response to external influences—ischemia, valvular insufficiency and stenosis, fibrillation, and hypertension—and may eventually progress to heart failure [10]. Further, in the cardiovascular system, histone modifications and chromatin remodeling are believed to modulate adaptive, as well as maladaptive, molecular pathways in HCM and heart failure [11]; as well, methylation of DNA has been responsible for the hypermutability of specific cardiac genes [12]. Additionally, investigations by Jirtle and Skinner [13] as well as Herceg and Vaissiere [14] allude to the potential interplay between environmental factors and the disease phenotype by epigenetic mechanisms. This has been successfully demonstrated in HCM in animal model studies, especially those originating from the Condorelli group [15-17]. Also, studies from the Hein group indicate strict epigenetic regulation during prenatal development and postnatal maturation, as well as in diseased human cardiomyocytes (CMCs) [18-20]. However, the knowledge about the impact of epigenetic alterations on the disease phenotype, specifically in HCM in human patients, is still very limited. This can be attributed to the limited availability of study specimens, because cardiac biopsy is a delicate procedure requiring cardiac catheterization.

Thanatology, which prioritizes the scientific study of death in the context of other fields of interest (ie, medicine, education, psychology, etc), has paved the way for genomic investigation of the pathological state; this is possible as formalin-fixed paraffin embedding of embalmed human cadavers preserves DNA, reduces the rate of decay, and promotes expansion of the available resource bank [21]. In fact, with the improvement in DNA isolation techniques, amplifiable DNA of high quality and quantity has been isolated from nonparaffin-embedded embalmed cadaver tissue [21].

This proposal is founded on the working hypothesis that the hydroxymethylome of cadaveric CMCs with HCM will exhibit epigenetic modification in the form of 5-hydroxymethylation of cytosine (5-hmC) in the genes, in line with observations from animal studies [19].

### Study Aims

**Figure 3:**
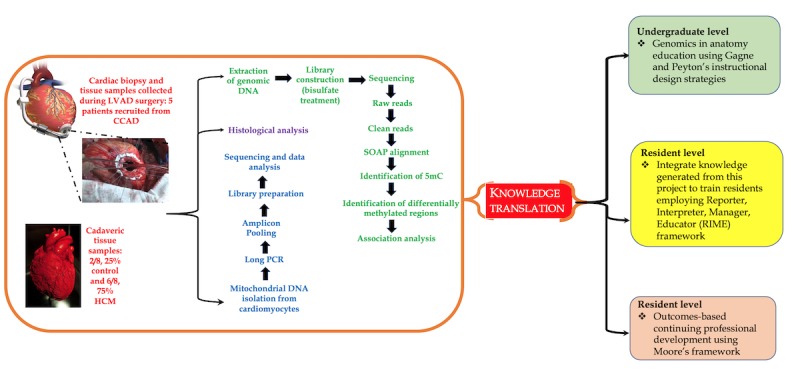
Summary of the proposed study; important steps are indicated (note: the results from this study will be used for competency-based medical education at different levels of training). 5mC: 5-methylation of cytosine; CCAD: Cleveland Clinic Abu Dhabi; HCM: hypertrophic cardiomyopathy; LVAD: left ventricular assist device; SOAP: short oligonucleotide alignment program.

In line with our working hypothesis, the *primary objective* of this study involves profiling the distribution of 5-hmC in the CMC genome in cardiac tissue obtained from cadavers and comparing them with the hydroxymethylome of biopsy specimens obtained from the apical part of the free left ventricle (LV); these were collected during *left ventricular assist device* surgery from HCM patients or cardiac transplant patients (control) undergoing cardiac catheterization using a standardized protocol. Additionally, as HCM is attributable to mutations in the mitochondrial DNA [22], we will compare the mitochondrial genome of cadaveric CMCs with that of biopsy specimens from HCM patients or cardiac transplant patients (control) undergoing cardiac catheterization. A summary of the study is shown in Figure 3.

The *secondary objective* of this study aims to integrate genomics education into medical education, specifically *anatomy* education, whereby we will blend Graham’s knowledge translation (KT) process [23] with instructional design models from Gagne and Peyton [24]. By doing so, we will strategize a lesson plan using the epigenetics of HCM as a case study to integrate *anatomy* with *genetics* and *biochemistry* into the core medical curriculum at Mohammed Bin Rashid University of Medicine and Health Sciences (MBRU).

In summary, this proposal aims to augment knowledge with regard to epigenetic regulation of HCM—one of the key cardiovascular concerns in the UAE—concomitantly strengthening medical education by integrating genomics education into *anatomy*. This will stimulate scientific inquiry among medical students, thereby providing KT, such that medical students can present new biomedical findings, correlating them with known clinical information about the patients’ diseases and traits.

## Methods

In order to address the *primary objective*, the following methodology will be implemented.

### Sample Collection

The total sample series will be obtained from two different types of tissue specimens, as follows: (1) cadaveric tissue and (2) biopsy specimens.

### Cadaveric Series

Normal and hypertrophic heart specimens will be obtained from 8 whole-body cadavers (2/8, 25% control and 6/8, 75% HCM), which have been donated for the promotion of teaching and research to MBRU. As indicated in the cadaver records, 6 cadavers presented with a previous history of pathological conditions causing HCM. All cadavers were commercially embalmed according to standard protocols [25].

### Dissection Protocol and Gross Anatomical Evaluation

The thickest heart slice will be selected and subjected to morphometric assessment. A digital caliper—Mitutoyo Digimatic Caliper, Model No. CD-8“ C (Mitutoyo Corporation)—will be used to measure the following parameters:

1. Thickness of the anterior, lateral, and posterior LV walls (ALV, LLV, and PLV, respectively).
2. Thickness of the anterior, lateral, and posterior right ventricle (RV) walls (ARV, LRV, and PRV, respectively).

Endocardial trabeculations, papillary muscles, and epicardial fat will be excluded from morphometric assessment.

In an effort to reduce intraobserver error, each parameter will be measured three times, from which a mean value will be determined. Clinical history with associated findings and demographic data (ie, age, race, sex, and nutritional status) pertaining to each cadaver will be documented. Ventricular wall thicknesses that exceeds 1.5 cm and 0.5 cm in the LV and RV, respectively, will be indicative of hypertrophy. These values will also be correlated with the weight of the heart and histological attributes of the CMCs to account for the effects of embalming, duration and method of storage, position of cadaver during storage, and rigor mortis. Heart specimens weighing more than 400 g will be described as hypertrophic.

### Recruitment Criteria: Myocardial Biopsy Series

Myocardial biopsy specimens will be obtained from the cardiothoracic and transplant units at the Cleveland Clinic in Abu Dhabi, UAE. Collection will be in accordance with the principles of the Declaration of Helsinki. All participants of the study must provide written informed consent, and the study will be appraised and approved by the relevant ethics committees. HCM will be diagnosed according to the 2014 European Society of Cardiology Guidelines [26]. Patients with valvular heart disease, history of myocarditis, regular alcohol consumption, or cardiotoxic chemotherapy will be excluded. The control biopsy specimens will be obtained from patients who had received heart transplants. To be included in the control cohort, patients would have received successful cardiac transplants more than 6 months previously, with normal systolic and diastolic function and no evidence of relevant vasculopathy as judged by coronary angiography. Furthermore, all control patients will have to exhibit freedom from relevant acute or chronic organ rejection. Details regarding processing of ventricular biopsy specimens are described below.

### Processing of Left Ventricle Biopsies

Tissue samples, measuring approximately 2 mm in length, will be extracted from the apical region of the LLV wall of 5 patients diagnosed with HCM and 3 cardiac transplant patients (control) undergoing cardiac catheterization, utilizing a standardized protocol used at the Cleveland Clinic in Abu Dhabi. Biopsies will be washed with NaCl (0.9%) and immediately transferred and stored in liquid nitrogen until DNA or RNA are extracted and histological analysis is pursued.

### Histological Analysis

#### Tissue Preparation

Cadaveric and biopsy specimens will be fixed in an embedding medium. Each specimen will then be dehydrated in a series of alcohol solutions of increasing concentration up to 100% alcohol. Washing will occur for 6-18 hours to remove water. Alcohol will be removed by infiltration of xylene. The specimen will then be placed in a cassette containing liquid paraffin. Once the melted paraffin has cooled and hardened into a mold, it will be trimmed into a suitably sized block. The block will be mounted in a specimen holder of the microtome sectioning machine. By careful rotary movement of the hand wheel, the block will cut thin sections in the form of a ribbon. Individual sections will be partitioned from the ribbon and placed in a water bath of 40°C. They will then be mounted on glass slides with albumin and allowed to dry.

#### Staining

The glass slide will be placed in xylene before it is passed through a series of solutions of decreasing alcohol concentration. Each glass slide will undergo routine staining with hematoxylin and eosin. Lee’s stain (ie, methylene blue and basic fuchsin) will then be employed to further highlight cytoplasmic organelles and enhance the contrast of the tissue.

The following connective tissue elements will be depicted: (1) nuclei in blue and (2) cytoplasm, mitochondria, and cilia in reddish-pink. The slide will then be passed through xylene to a nonaqueous mounting medium. A coverslip will be placed over the specimen on the slide to attain permanent preparation.

#### Semiquantitative Analysis

A total of three field areas per slide will be examined. Cross-sectional area, length, and width of CMCs will be quantified. In addition, organization of sarcomeres, mitochondrial size, and arrangement of cristae will be described.

#### Level of Significance

Statistical analysis will be performed using SPSS Statistics for Windows, version 24.0 (IBM Corp). The means and frequencies of the continuous and categorical variables, respectively, will be compared for difference or equivalence between parameters and demographically relevant factors.*P*P s of less than .05 will be considered statistically significant.

### Profiling, Sequencing, and Analysis of the Hydroxymethylome

#### Extraction of DNA

DNA will be collected from cadaveric tissues according to the method of Gielda and Rigg, which involves modification of existing techniques of tissue disruption, combined with phenol-chloroform treatment [21]. For biopsy samples, we will use the protocol of Haas et al [27].

Total DNA will be extracted from CMCs from 6 cadaveric hearts diagnosed with HCM, 2 normal cadaveric hearts (control), and biopsy specimens obtained from the apical part of the free LV wall from 5 HCM patients. In addition, DNA will be extracted from the CMCs of 3 cardiac transplant patients (control) undergoing cardiac catheterization. DNA will be enriched for 5-hmC using a biotin-based streptavidin pull-down technique—Hydroxymethyl Collector, Active Motif (Epigenie)—originally described by Song et al [28,29]. The rationale behind employing Song’s technique is that it has been successfully used in other studies [30,31].

#### Sequencing and Analysis of the Hydroxymethylome

Libraries will be prepared using 500 ng of 5-hmC-enriched DNA using the NEBNext Kit (New England Biolabs) and will be sequenced on the BGISEQ-500 platform (Beijing Genomics Institute, BGI), carried out by the BGI epigenetic sequencing service [32], where the corresponding author has collaborative projects.

The BGISEQ-500 generates approximately 500 Gb of sequence data per flow cell, or about 62 Gb per lane. Therefore, a single human genome library can be run across two lanes of the eight-lane flow cell to generate approximately 120 Gb of data per sample. Additional sequencing will be pursued for higher coverage.

Hydroxymethylation analysis will be performed using the Bismark online module for reading bisulfite sequence maps. In summary, FASTQ files will be quality filtered and adapter sequences will be trimmed using the Trimmomatic tool [33]. A bisulfite-converted human genome (HG19) reference genome file will be generated using Bowtie [34], and the EpiGnome (Epicentre) library sequence data will be aligned to the reference genome.

Hydroxymethylation information will be extracted from the output *.sam file, and genome tracks will be the output for visualization and reporting of downstream differential methylation calculations.

The hydroxymethylation extraction report should show minimal bias. A perfectly unbiased sequencing run would be a horizontal line. Visualization of hydroxymethylated sites of the genome will be performed using the Integrative Genomics Viewer (Broad Institute) [35].

To identify regions that gained or lost 5-hmC at specific differentially hydroxymethylated regions (DhMRs), the normal samples will be set as the control group and the HCM samples as the treated group, and the diffReps program [36] will be used with default settings. The diffReps program normalizes each sample by removing regions of low read counts and then calculates a normalization ratio for each sample based on the remaining reads. The medians of the ratios will then be used as normalization factors. To assess differential sites, diffReps uses a negative binomial test on sliding windows and the significant windows will be selected by a predefined cutoff (*P*<.001). The significant windows that overlap with each other will then be merged, and the differential sites will be used to perform the statistical tests again. The*P*P for each differential site and the best*P*P for the sliding window within each differential site will be documented and reported. Subsequently, DhMRs will be classified by diffReps into genomic locations and annotated using the human (HG19) reference genome.

### Mitochondrial DNA Isolation

Mitochondrial DNA will be isolated from cadaveric and biopsy samples, for both HCM and control groups, employing the method of Quispe-Tintaya et al, as this technique exhibits an approximately 2000-fold enrichment of mitochondrial DNA in comparison to commercial kits and is also relatively cheaper [37]. Briefly, the process has two steps: (1) extraction of a mitochondrial DNA-enriched fraction employing a common plasmid miniprep kit and (2) additional purification of mitochondrial DNA using the Agencourt AMPure XP system (Beckman Coulter).

### Library Preparation and Sequence Analysis of the Mitochondrial Genome

**Figure 4:**
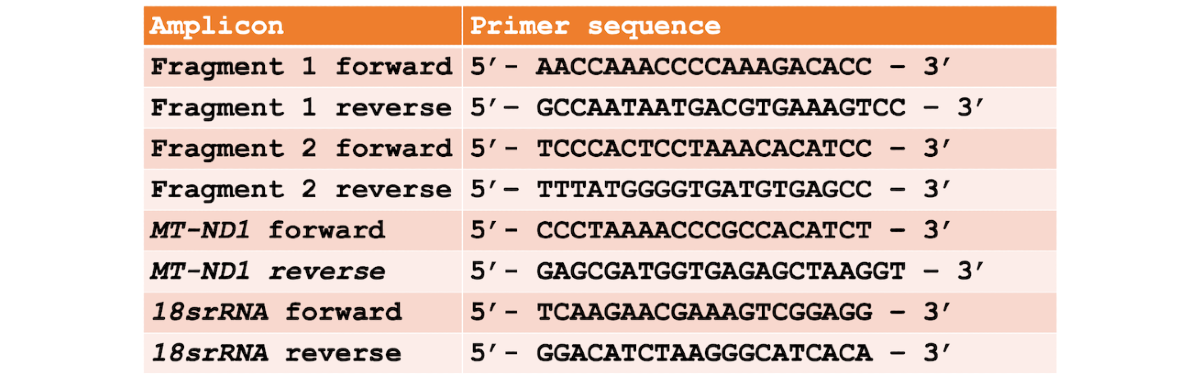
Sequence of primers that will be used to amplify cardiomyocyte (CMC) mitochondrial DNA fragment 1 (9289 bp in length) and fragment 2 (7626 bp in length). 18srRNA: 18S ribosomal RNA; MT-ND1: mitochondrial NADH-ubiquinone oxidoreductase chain 1.

The CMC mitochondrial DNA will be amplified in two fragments using high-fidelity, long-distance polymerase chain reaction (PCR) with a proofreading polymerase—LA Taq DNA polymerase (TaKaRa)—as described by Tang et al [38]. The primers that will be used to amplify cardiac CMC mitochondrial DNA fragment 1 (9289 bp in length) and fragment 2 (7626 bp in length) are shown in Figure 4. The amplified fragments will be used for preparation and sequencing.

Complementary DNA (cDNA) libraries will be prepared with Nextera sample preparation kits (Illumina). These libraries will be quantified using a PicoGreen assay (Invitrogen) and a Kapa quantitative PCR (qPCR) library quantification kit (Kapa Biosystems). Size and quality of cDNA libraries will be confirmed using an Agilent Bioanalyzer 2100 DNA high-sensitivity chip. The cDNA libraries will then be pooled based on qPCR values. cDNA products will be sequenced in rapid mode using the BGISEQ-500 platform.

Paired-sequence reads will be demultiplexed using bcl2fastq, version 1.8.4 (Illumina). Reads will then be mapped to the reference mitochondrial genome—NC_012920.1 (version GI: 251831106)—and will undergo variant calling using the CLC Genomics Workbench, version 8.0.1 (QIAGEN), alignment tool. This will require a minimum coverage of 1000× and a minimum frequency of 1.00 × 10^-6^. An average of 7.50 million reads will be mapped per sample. Sequencing data will then be compiled into Microsoft Excel spreadsheet form for analysis. Data in the form of mitochondrial DNA alterations—mutations in specific genes and single-nucleotide polymorphisms (SNPs) at specific loci—between the control and HCM samples will be analyzed using the chi-square test and the Fisher exact test.*P*P s of less than .05 will be considered statistically significant.

### Knowledge Translation

**Figure 5:**
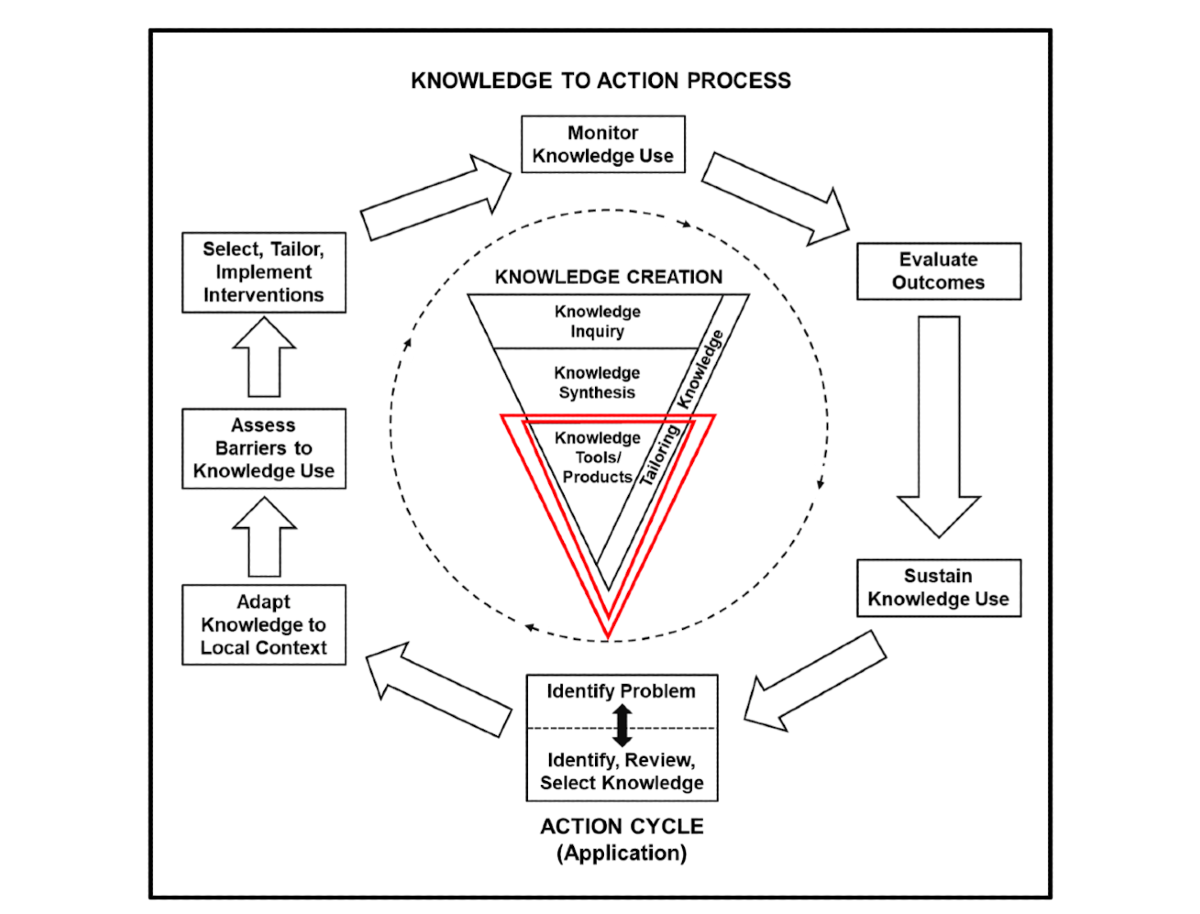
Graham's knowledge translation process (Straus et al, 2011). The central idea of this process is to create knowledge through diverse knowledge tools, which may be in the form of research, that could create new knowledge.

In order to address the *secondary objective*, we will employ Graham’s KT process (see Figure 5). KT is a dynamic and iterative process that includes synthesis, dissemination, exchange, and ethically sound application of knowledge to improve the health of Emiratis, provide more effective health services and products, and strengthen the health care system [23].

**Figure 6:**
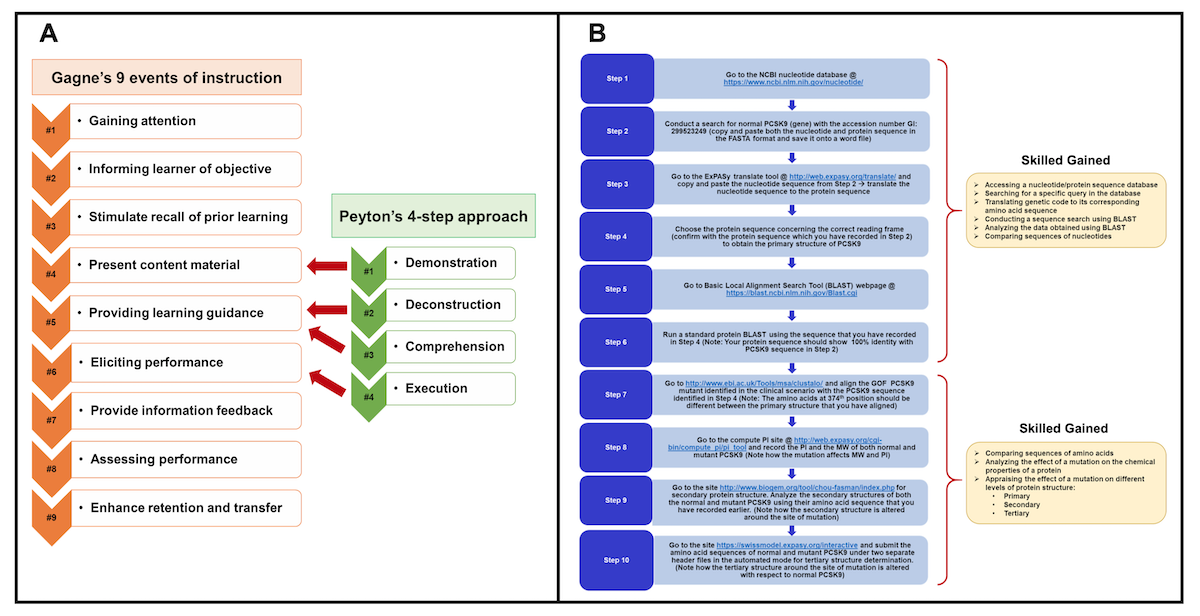
The framework for knowledge translation to be implemented in this study, elaborated using a vignette of autosomal dominant familial hypercholesterolemia. A. Description of the blended lesson plan: Gagne's events of instruction and Peyton's approach. B. The sequential steps of the lesson plan and skills gained. ExPASy: Expert Protein-Analysis System of the Swiss Institute of Bioinformatics; GOF: gain of function; NCBI: National Center for Biotechnology Information; PCSK9: proprotein convertase subtilisin/kexin type 9; PI: isoelectric point; MW: molecular weight.

In order to integrate *genomics* into *anatomy* education, we will use a blended approach where we will blend Gagne’s approach with Peyton’s approach [24]. A successful implementation of such KT using these approaches is explained using a scenario of familial hypercholesterolemia [39-41], which is the key research interest of the corresponding author. Here, data obtained from a project on familial hypercholesterolemia is used to augment knowledge regarding *bioinformatics* in undergraduate medical education. Data shown here are from an article by Tambi et al, published in JMIR Medical Education (see Figure 6) [24].

**Figure 7:**
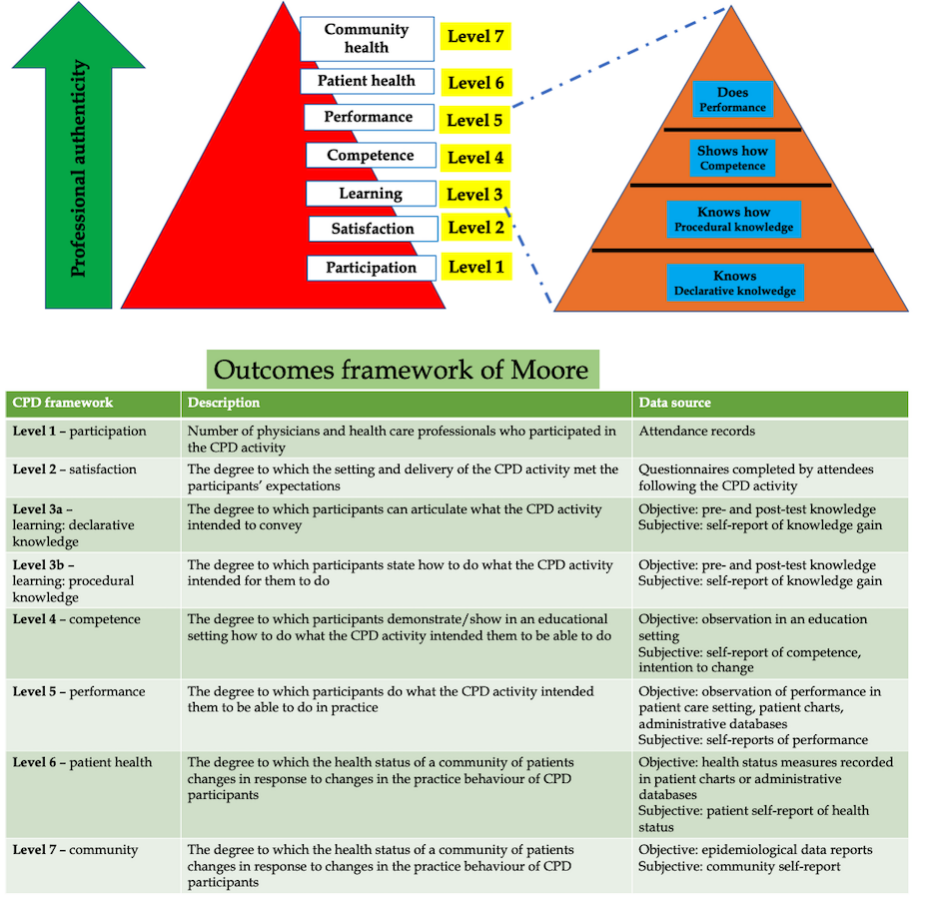
Framework for outcomes-based continuing professional development (CPD) to be used in the proposed research.

The proposed research will generate new knowledge with regard to HCM. We aim to add this to the existing knowledge available in the literature and adapt it to suit the needs of medical education in the UAE by assessing barriers to knowledge use (see Figure 5). We will do this via the following proposed KT strategies:

1. At the *undergraduate medical student* level, integration of *genomics* into anatomy education will be pursued by the implementation of instructional design strategies, specifically by blending Gagne’s and Peyton’s approaches (see Figure 6) [24].
2. At the *resident* level, KT will involve Pangaro’s *Reporter, Interpreter, Manager, Educator* (RIME) framework [42], as this model has particular merit for providing feedback to residents.
3. At the *practicing physician* level (noncardiologists), KT will be facilitated through outcomes-based continuing professional development devised by Moore (see Figure 7) [43,44].

## Results

**Figure 8:**
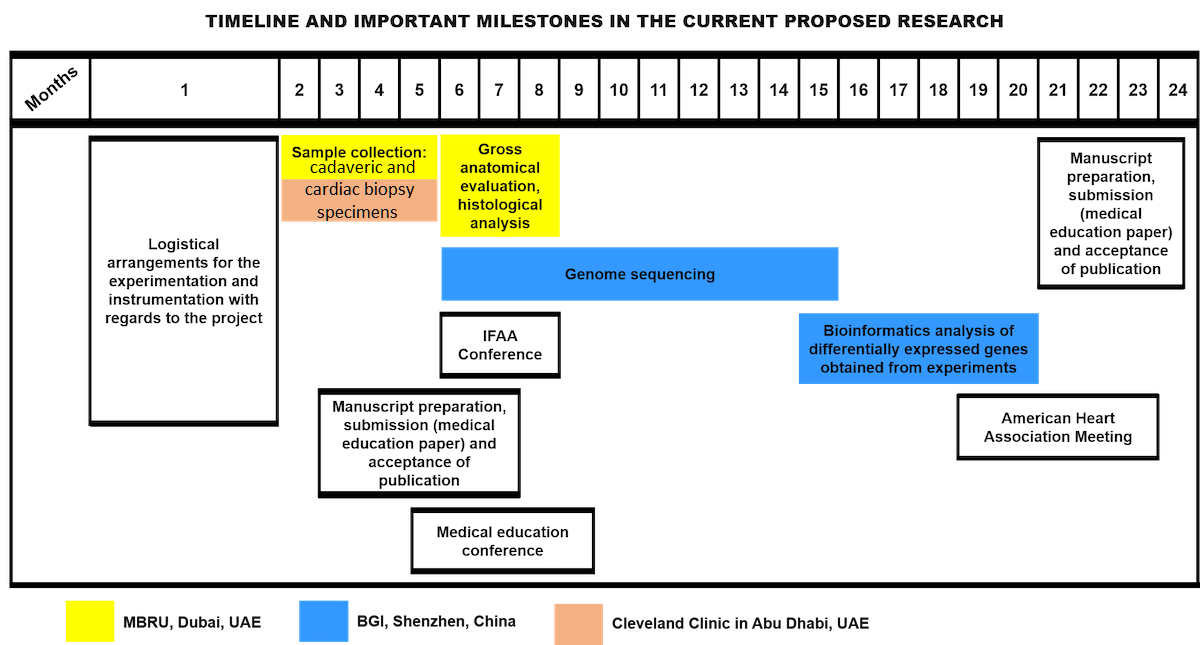
Timeline and key milestones for the study; the sites where the research will be conducted are also indicated. BGI: Beijing Genomics Institute; IFAA: International Federation of Associations of Anatomists; MBRU: Mohammed Bin Rashid University of Medicine and Health Sciences; UAE: United Arab Emirates.

This study is at the protocol-development stage. The validated questionnaires have been identified in relation to the objectives. The MBRU and the Cleveland Clinic Abu Dhabi Institutional Review Boards (IRB) are reviewing this study. Further clarification and information can be obtained from the MBRU IRB. There is funding in place for this study (MBRU-CM-RG2019-08). Currently, we are in the process of standardizing the protocols with respect to the various molecular techniques to be employed during the course of the study. The total duration of the proposed research is 24 months, with a provision for 6 months of a no-cost extension. Key project milestones and the timeline are shown in Figure 8.

## Discussion

### Overview

To the best of our knowledge, this is the first study exploring the correlation between the HCM phenotype and alterations in mitochondrial DNA. If a correlation is observed, then a study in a larger HCM cohort is warranted.

Additionally, we believe that specific adjustments will be required in the protocol by Quispe-Tintaya et al [37] when isolating mitochondrial DNA from cadaveric samples. This will provide a standardized protocol for such a procedure, benefitting medicine as well as forensic research.

The problem of nuclear copies of mitochondrial DNA (NUMTs) is often encountered while sequencing mitochondrial DNA. Double bands in PCR results or double peaks in mitochondrial DNA sequences indicate NUMTs, however, the case of mitochondrial DNA heteroplasmy has to be ruled out first. In order to avoid NUMTs, we are using mitochondrial DNA-specific primers and a rigorous mitochondrial DNA purification process. Furthermore, this work will be pursued at BGI [45], where the protocol for the required experimentation is standardized [38].

For the *secondary objective*, data will be collected through semistructured interviews regarding participants’ use of genomics in their understanding of HCM, as well as the ability of different frameworks to raise awareness regarding the importance of HCM with regard to the Emirati population.

We believe that successful implementation of KT frameworks will support the *primary objective*, as increased participation in these frameworks will ensure successful dissemination of sequence analysis—in the form of undergraduate students participating in the process—and obtainment of specimens—in the form of resident and physician participation.

### Conclusions

The spectrum of CVDs has recently received significant focus from the public health sector in the UAE. While HCM is a common familial heart disease, it is now considered to be one of four CVDs contributing to the sudden increase in the mortality rate of young Emiratis in the UAE. Incorporating artificial intelligence to identify the epigenetic risk factors associated with HCM will promote accurate diagnosis and lead to the development of improved management plans, hence, positive patient outcomes. Furthermore, integration of these findings into the instructional design of undergraduate, postgraduate, and continuous professional development medical curricula will further contribute to the body of knowledge regarding HCM. In summary, this proposal aims to augment knowledge with regard to epigenetic regulation of HCM. This concomitantly strengthens medical education by integrating *genomics* education into *anatomy* to stimulate scientific inquiry among medical students. KT is thereby provided, such that medical students can present new biomedical findings, correlating them with known clinical information about patients’ diseases and traits.

Firstly, while this study will include a small cohort of specimens due to tissue accessibility, it will begin to answer the question, “Is there a correlation between epigenetic modification of the genome and HCM phenotype?” and will allude to whether mitochondrial DNA alterations have detrimental consequences with regard to CMC structure and function. If the investigational approaches employed in this study hint toward a positive correlation, future study of the hydroxymethylome in a larger cohort of HCM patients will be warranted.

Secondly, as cadaver and patient records will provide information regarding other comorbidities (eg, diabetes, metabolic syndrome, and dyslipidemia), the underlying effect of these conditions on the severity of HCM may be elucidated, which, like above, will require confirmation in a larger cohort of patients with HCM. This study will also pave the way to design the strategy of integrating genomics education into anatomy teaching, as well as KT through different frameworks at different levels and competence of medical training. The success of this integration may be evaluated via different models of feedback, such as that of Pendleton [46], and structured questionnaires.

## Appendix

Multimedia Appendix 1 Peer-reviewer report from MBRU College of Medicine.

## Abbreviations

5-hmC: 5-hydroxymethylation of cytosine
ALV: anterior left ventricle
ARV: anterior right ventricle
BGI: Beijing Genomics Institute
cDNA: complementary DNA
CMC: cardiomyocyte
CVD: cardiovascular disease
DhMR: differentially hydroxymethylated region
HCM: hypertrophic cardiomyopathy
IRB: Institutional Review Board
KT: knowledge translation
LLV: lateral left ventricle
LRV: lateral right ventricle
LV: left ventricle
MBRU: Mohammed Bin Rashid University of Medicine and Health Sciences
MYH7: myosin heavy chain beta isoform
NUMT: nuclear copy of mitochondrial DNA
PCR: polymerase chain reaction
PLV: posterior left ventricle
PRV: posterior right ventricle
qPCR: quantitative polymerase chain reaction
RIME: Reporter, Interpreter, Manager, Educator
RV: right ventricle
SNP: single-nucleotide polymorphism
UAE: United Arab Emirates
