# Supplementary material for: Thanatogenomic Investigation of the Hydroxymethylome and Mitochondrial Genome of Cadaveric Cardiomyocytes: Proposal for a Proof-of-Concept Study
Source: JMIR Res Protoc. 2020 Mar 5;9(3):e17241. doi: 10.2196/17241 (PMC7082735; doi:10.2196/17241)
Supplement: Multimedia Appendix 1 [file resprot_v9i3e17241_app1.pdf]

Reviewer 1 Comments

SUMMARY AND RECOMMENDATIONS:

Please supply comments that provide an overall assessment of the proposal, emphasizing: Strengths and weaknesses that most influence your overall funding recommendation. Please note specific areas of the proposal that should be revised to strengthen the proposal.

1. The project aims are too discrete and there is no apparent connection between each other. For example, objective 2 is to profile 5-hmC methylation, whereas objective 4 aimed to profile mitochondrial genomes. The proposal expanded on experimental protocols (b but lacks rational on explaining the objectives. For example, it is not clear at all why project aims to correlate mitochondrial genome (mtDNA) with HCM phenotype and how 5-hmC fits into explaining disease risk.
2. The project did not specify why it is not conducting genome or targeted sequencing since there are a lot of genes that are well known to be strongly associated with HCM (as written in the background).
3. One of the major weaknesses of the project is the quality of methylation and DNA degradation. It is well known that long DNA extraction interval from postmortem tissue is not a great source to profile methylation. This might impact the overall outcome of the project.
4. Sample size is extremely small and might not have enough statistical power to infer reliable conclusion that is reproducible.
5. The coined term ‘THANOTOGENOMICS’ and its given definition lacks correlation due to the fact that the project will conduct bisulphite sequencing to map out methylation patterns, not genomes. THANOTOEPIGENOMICS would be a better term in correlation with the experiments.

SUMMARY AND RECOMMENDATIONS:

Please supply comments that provide an overall assessment of the proposal, emphasizing: Strengths and weaknesses that most influence your overall funding recommendation. Please note specific areas of the proposal that should be revised to strengthen the proposal.

The project is feasible. But the applicant needs to have more focused aims. It is not clear whether the project is aimed at one or all of the different types of HCM (Familial; Acquired or mitochondrial).
